# Molecular reprogramming in cutaneous melanoma: the central role of G9a and EZH2 methyltransferase inhibitors in tumor plasticity, immune evasion, and therapeutic resistance

**DOI:** 10.3389/fphar.2026.1879451

**Published:** 2026-09-03

**Authors:** Melissa Yoshimi Sakamoto Maeda Nisimoto, Daniel Arcuschin de Oliveira, Heitor Raia Bottura, Wagner Robson Germano Sousa, Gabrielle do Nascimento Sividanes, Amanda Fáris Marques, Jaciara Moreira Sodré Hunnicutt, Alexandre Giannecchini Romagnolo, Maria Clara Ferreira Vivi, Beatriz Linhares Gorini, Francisco Macedo Paschoal, Miguel Sabino Neto, Miriam Galvonas Jasiulionis, Luciana Cavalheiro Marti, Renato Santos de Oliveira Filho

**Affiliations:** 1 Melanoma and Skin Tumors Sector, Plastic Surgery Discipline, Escola Paulista de Medicina/Universidade Federal de São Paulo EPM/UNIFESP), SãoPaulo, Brazil; 2 Department of Pharmacology, Escola Paulista de Medicina, Universidade Federal de São Paulo EPM/UNIFESP, SãoPaulo, Brazil; 3 Experimental Research, Instituto Israelita de Ensino e Pesquisa Albert Einstein, Hospital Israelita Albert Einstein, SãoPaulo, Brazil; 4 Dermatology Discipline, FMABC University Center, SãoPaulo, Brazil

**Keywords:** EZH2, G9a, melanoma, metastasis, methyltransferase

## Abstract

Cutaneous melanoma remains a leading cause of skin cancer-related mortality despite significant therapeutic advances, with treatment resistance and immune evasion representing major clinical challenges. Epigenetic dysregulation, particularly through the histone methyltransferases G9a (EHMT2) and Enhancer of Zeste Homolog 2 (EZH2), plays a pivotal role in melanoma progression, therapeutic resistance, and immune escape. G9a catalyzes the deposition of H3K9me2, whereas EZH2, the catalytic subunit of the Polycomb Repressive Complex 2 (PRC2), mediates H3K27me3 deposition, thereby establishing repressive chromatin states that silence tumor suppressor genes and immune-related pathways. These enzymes are frequently overexpressed in melanoma and cooperate to maintain dedifferentiated and invasive tumor phenotypes characterized by enhanced plasticity and reduced immunogenicity. Acral melanoma, a distinct subtype with a low ultraviolet (UV) mutational burden, appears to be particularly dependent on epigenetic reprogramming and exhibits an immunologically “cold” tumor microenvironment that is associated with poor responses to current therapies. Preclinical studies demonstrate that pharmacological inhibition of G9a with compounds such as UNC0642 and EZH2 with agents including tazemetostat can restore the expression of silenced genes, enhance antigen presentation, and improve tumor immunogenicity. Importantly, the functional crosstalk between G9a and EZH2 creates redundant repressive mechanisms that may limit the efficacy of single-agent therapies supporting the development of combination strategies targeting both pathways. Dual inhibition has shown promise in pre-clinical models by converting immune-excluded “cold” tumors into immune-responsive “hot” tumors that are more susceptible to checkpoint blockade and adoptive cell therapies. While EZH2 inhibitors have progressed to clinical trials in various solid tumors, melanoma-specific studies remain limited despite strong biological rationale. This review synthesizes the current understanding of G9a and EZH2 biology in melanoma, evaluates therapeutic targeting strategies, and highlights the potential for epigenetic reprogramming to overcome treatment resistance and enhance immunotherapy responses in this aggressive malignancy.

## 1 Introduction

Cutaneous melanoma is an aggressive malignancy arising from the malignant transformation of melanocytes. Although it represents a relatively small proportion of skin cancers, melanoma accounts for most skin cancer–related deaths worldwide, and its incidence has steadily increased in recent decades, driven by multifactorial risk factors, including rising levels of ultraviolet (UV) radiation exposure observed over recent decades, environmental and climate-related influences, genetic susceptibility, and host-related determinants (Arnold et al., 2022). Epidemiological projections indicate a continued rise in both incidence and mortality if current trends persist (Arnold et al., 2022). Biologically, cutaneous melanoma is characterized by a high tumor mutational burden predominantly associated with UV-induced DNA damage, frequent alterations in key oncogenes, and substantial phenotypic and molecular heterogeneity (Caraviello et al., 2025). These features contribute to tumor aggressiveness and influence therapeutic responses, which currently include surgical resection in early stages and systemic approaches, such as immune checkpoint blockade, targeted therapies and adoptive cellular therapy in advanced disease. Despite these advances, primary and acquired resistance remain common, underscoring the need for new therapeutic strategies.

Epigenetic dysregulation and dynamic chromatin modifications modulate gene expression programs involved in tumor progression, immune escape, and therapeutic resistance. Histone methyltransferases such as Euchromatic Histone Lysine Methyltransferase 2 (EHMT2), also known as G9a, and Enhancer of Zeste Homolog 2 (EZH2) catalyze the repressive histone marks H3K9me2 and H3K27me3, respectively, resulting in transcriptional silencing of tumor suppressor genes and genes essential for antitumor immune responses (Flesher and Fisher, 2021). Given the reversibility of these epigenetic modifications, selective inhibitors targeting G9a and EZH2 have attracted increasing interest as therapeutic candidates, mainly in combination with established treatments.

Among melanoma subtypes, acral melanoma (AM) represents a clinicopathologically and molecularly distinct entity. Typically arising on glabrous skin, AM is characterized by a low UV mutational signature and a greater dependence on epigenetic reprogramming (Carvalho et al., 2023; Wang et al., 2025; Minowa et al., 2024). In contrast to conventional cutaneous melanoma, AM frequently exhibits an immunologically “cold” tumor microenvironment, marked by reduced immune activation, diminished inflammatory signatures, reduced expression of BRAF mutations, and poorer responses to immunotherapies and targeted therapies (Carvalho et al., 2023; Wang et al., 2025; Minowa et al., 2024). These features suggest that epigenetic mechanisms may play a particularly central role in shaping tumor plasticity and immune escape in this subtype.

Two major epigenetic regulatory axes contribute to melanoma progression: the EHMT2–H3K9me2–HP1 pathway and the PRC2–EZH2–H3K27me3 axis. G9a mediates deposition of H3K9me2, recruiting Heterochromatin Protein 1 (HP1) to promote chromatin compaction and transcriptional silencing. Similarly, Polycomb Repressive Complex 2 (PRC2), through EZH2 and its cofactors, Embryonic Ectoderm Development (EED) and Suppressor of Zeste 12 homolog (SUZ12), establishes H3K27me3-mediated repression (Kim and Tsao, 2025; Giunta et al., 2021; Lim et al., 2023). Hyperactivation of these pathways sustains dedifferentiation states and tumor plasticity while promoting immune evasion through suppression of antigen presentation pathways and chemokine expression critical for immune cell recruitment (Kim and Tsao, 2025; Giunta et al., 2021; Lim et al., 2023).

Preclinical and translational evidence supports these enzymes as actionable therapeutic targets. Pharmacological inhibition of G9a has been shown to enhance responses to immune checkpoint blockade (Kelly et al., 2021). One such G9a inhibitor, UNC0646, induces melanoma cell death and promotes measurable epigenetic reprogramming, supporting its potential not only as a therapeutic agent but also as part of a biomarker-driven strategy to identify patient subgroups most likely to respond (Filiú-Braga et al., 2024).

Furthermore, resistance to immunotherapy in melanoma has been linked to epigenetically driven transcriptional programs, providing a rationale for integrating markers such as H3K27me3 and PRC2 activity into translational frameworks (Li et al., 2024). Hyperactive epigenetic states correlate with reduced CD8^+^ T-cell infiltration and lower PD-L1 expression, reinforcing the immunosuppressive phenotype observed particularly in acral melanoma (Carvalho et al., 2023; Wang et al., 2025; Kim and Tsao, 2025).

Importantly, PRC2-mediated repression operates within a dynamic chromatin landscape influenced by interactions with remodeling complexes such as the SWItch/Sucrose Non-Fermentable (SWI/SNF), including key subunits SWI/SNF-related matrix-associated actin-dependent regulator of chromatin subfamily B member 1 (SMARCB1) and subfamily A member 4 (SMARCA4). Alterations in these components frequently arise during melanoma evolution and modulate tumor dependence on PRC2 activity. The functional antagonism between chromatin remodeling and Polycomb-mediated condensation creates synthetic vulnerabilities that may inform future molecular and epigenetic stratification strategies (Sisakht et al., 2023; Tigu et al., 2025).

## 2 Cutaneous melanoma: clinical and molecular overview

### 2.1 Epidemiology and clinical burden

Despite major therapeutic advances for melanoma, advanced-stage melanoma continues to impose a substantial clinical burden, particularly in patients who develop metastatic disease or relapse after systemic treatment.

Melanoma is molecularly heterogeneous and can be broadly classified into major genomic subtypes based on recurrent driver alterations including v-Raf murine sarcoma viral oncogene homolog B1 (BRAF)-mutant, Neuroblastoma RAS viral oncogene homolog (NRAS) mutant, Neurofibromin 1 (NF1)-mutant and triple wild-type melanoma. These subgroups differ in oncogenic signaling dependencies, tumor biology, the immune landscape, and therapeutic vulnerabilities. Activating BRAF mutations, most commonly BRAF^V600E^, drive constitutive mitogen-activated protein kinase (MAPK) signaling and occur in approximately half of cutaneous melanomas, whereas *NRAS* mutations similarly promote MAPK and phosphoinositide 3-kinase (PI3K) pathway activation. *NF1*-mutant melanomas are characterized by dysregulated Rat sarcoma (RAS) signaling and increased genomic complexity, while triple wild-type tumors often harbor alternative drivers, including KIT proto-oncogene receptor tyrosine kinase, G protein subunit alpha q (GNAQ)/G protein subunit alpha 11 (GNA11), or structural and epigenetic alterations, underscoring the biological diversity of this disease (Bastian, 2014; TCGA, 2015).

### 2.2 Epigenetic dysregulation in melanoma

**FIGURE 1:**
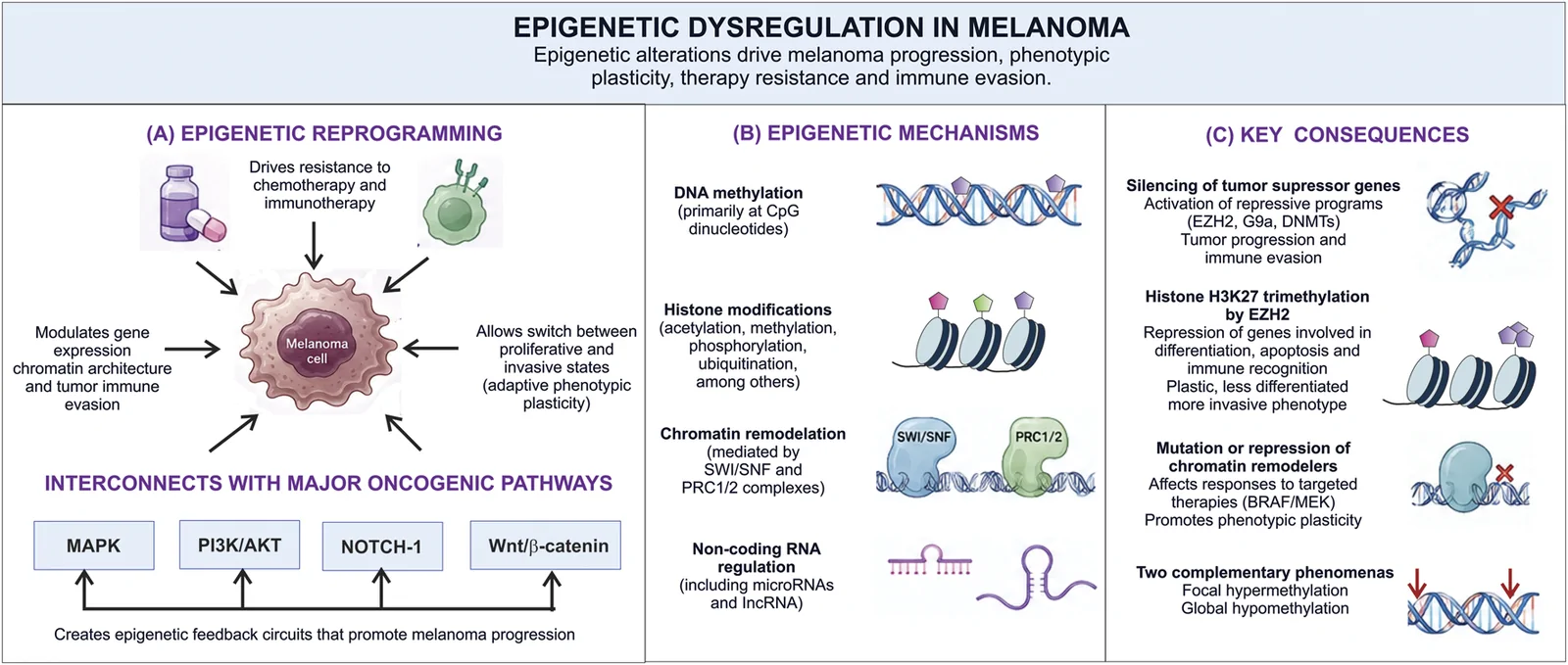
Epigenetic dysregulation in cutaneous melanoma. Schematic overview of the principal epigenetic mechanisms driving melanoma progression, phenotypic plasticity, immune evasion, and therapeutic resistance. **(A)** Epigenetic reprogramming promotes adaptive phenotypic switching through transcriptional regulation and interaction with major oncogenic pathways, including MAPK, PI3K/AKT, NOTCH, and Wnt/β-catenin. **(B)** Major epigenetic regulatory mechanisms include DNA methylation, histone modifications, ATP-dependent chromatin remodeling, and non-coding RNAs. **(C)** These alterations promote tumor suppressor gene silencing, dedifferentiation, immune escape, and resistance to targeted therapy and immunotherapy through coordinated actions of epigenetic regulators, including G9a, EZH2, DNMTs, and chromatin-remodeling complexes, together with promoter hypermethylation and global DNA hypomethylation.

Epigenetics refers to heritable and reversible modifications in gene expression without alterations in the DNA sequence. These modifications determine which genes are “turned on” or “turned off,” thereby controlling cellular identity and behavior (Figure 1A). The main epigenetic mechanisms include DNA methylation (primarily at CpG dinucleotides), histone modifications (acetylation, methylation, phosphorylation, ubiquitination and others), chromatin remodeling (mediated by SWI/SNF and PRC1/2 complexes), and regulation by non-coding RNAs (including microRNA and lncRNA) (Skourti and Dhillon, 2022).

Epigenetic reprogramming drives resistance to chemotherapy and immunotherapy by modulating gene expression, chromatin architecture, and tumor immune evasion (Walker et al., 2023). This process interconnects with major oncogenic pathways in melanoma, including MAPK, phosphoinositide 3-kinase/protein kinase B (PI3K/AKT), NOTCH-1 signaling, and Wingless/Integrated-beta-catenin (Wnt/β-catenin). This interplay creates epigenetic feedback circuits (Figure 1A) that allow melanoma cells to switch between proliferative and invasive states, a phenomenon known as adaptive phenotypic plasticity. The reversibility of epigenetic alterations provides new avenues for combinatorial therapeutic strategies (Parab et al., 2023).

Epigenetics acts as a bridge between the genome and the tumor microenvironment, controlling cellular plasticity, adaptation, and resistance. The silencing of tumor suppressor genes and the activation of repressive epigenetic programs (mediated by EZH2, G9a, and DNMTs) are key events in tumor progression and immune evasion (Figure 1B). Combined epigenetic therapies hold promise for overcoming resistance to immunotherapy and targeted therapies (Tigu et al., 2025).

The epigenetic biology of melanoma involves two complementary phenomena: the inhibition of tumor suppressor genes, leading to the silencing of regulatory genes, genomic instability, tumor progression, immune escape, and global hypomethylation, resulting in the reactivation of transposable elements with mutagenic insertions, which contributes to chromosomal instability and increased intratumoral heterogeneity (Figure 1C). Thus, while focal hypermethylation silences tumor suppressor genes, diffuse hypomethylation promotes genomic instability and tumor plasticity (Vitale et al., 2026).

Histones undergo post-translational modifications that alter chromatin compaction. Trimethylation of histone H3 at lysine 27, catalyzed by EZH2, is one of the most relevant epigenetic marks in metastatic melanoma (Figure 1C). Overexpression of EZH2 represses genes involved in melanocytic differentiation (*MITF, MLANA*), apoptosis (*BIM*), and immune recognition (*HLA-A, HLA-B*), thereby maintaining a more “plastic,” less differentiated, and more invasive phenotype that is resistant to immunotherapy (Hoffmann et al., 2020).

Enzymes such as BRG1/SMARCA4 (SWI/SNF) and ARID1A/B regulate chromatin accessibility and remodeling. Mutations or repression of these genes affect the response to targeted therapies (BRAF/MEK) and promote phenotypic plasticity, allowing tumor subpopulations to survive under therapeutic pressure (Sisakht et al., 2023).

Epigenetic drugs are agents that modulate epigenetic mechanisms to reverse aberrant gene regulation patterns that sustain tumor transformation and progression. Important classes include DNA methyltransferase inhibitors (azacitidine, decitabine), histone deacetylase inhibitors, inhibitors of histone “writers” and “erasers” (G9a inhibitors such as UNC0642; EZH2 inhibitors such as tazemetostat), and chromatin remodeler inhibitors (targeting the SWI/SNF). These drugs act not only by directly controlling tumor proliferation but also by modulating the immune microenvironment, DNA repair, and cellular differentiation, making epigenetic agents promising partners for targeted therapies and immunotherapy (Emran et al., 2019; Sigalotti et al., 2010).

## 3 Histone methyltransferases in cancer

### 3.1 Overview of histone lysine methylation

Histone lysine methylation is a central epigenetic mechanism that regulates chromatin architecture and gene expression, with profound implications for development, cell identity, and tumorigenesis. This post-translational modification is catalyzed by histone lysine methyltransferases (KMTs), which transfer methyl groups to lysine residues on histone tails, thereby generating epigenetic marks associated with either transcriptional activation or repression, depending on the target residue and methylation state (Tachibana et al., 2005).

Among these modifications, methylation of histone H3 lysine 9 (H3K9) and histone H3 lysine 27 (H3K27) represents two of the most prominent repressive chromatin marks in cancer. H3K9 methylation, deposited by EHMT2/G9a and Suppressor of Variegation 3–9 Homolog 1 (SUV39H1), is commonly associated with heterochromatin formation, chromatin compaction, and stable transcriptional silencing (Tachibana et al., 2005). Dimethylation of H3K9 (H3K9me2) is linked to facultative heterochromatin and the widespread repression of lineage-specific and tumor suppressor genes, whereas trimethylation (H3K9me3) is predominantly associated with constitutive heterochromatin, genomic integrity and genome stability (Allis and Jenuwein, 2016).

By contrast, H3K27 methylation, primarily catalyzed by EZH/PRC2, he catalytic subunit of Polycomb Repressive Complex 2 (PRC2) mediates transcriptional silencing through repression. The trimethylated H3K27 (H3K27me3) is a hallmark of facultative heterochromatin and plays critical roles in developmental gene repression, cellular plasticity, and maintenance of undifferentiated cellular states. In cancer, aberrant accumulation of both H3K9me2 and H3K27me3 contributes to epigenetic silencing of tumor suppressive, immune-related, and differentiation-associated genes (Allis and Jenuwein, 2016).

Histone lysine residues can exist in mono- (me1), di- (me2), or tri-methylated (me3) states, each conferring distinct functional outcomes. These methylation states are not merely quantitative intermediates but constitute qualitatively different regulatory signals, recognized by specialized reader proteins that recruit chromatin remodeling and transcriptional machinery. For example, H3K9me2 serves as a docking site for HP1 family proteins promoting chromatin compaction, whereas H3K27me3 recruits of Polycomb Repressive Complex 1 (PRC1), reinforcing transcriptional repression through chromatin condensation and histone ubiquitination.

Among the numerous KMTs identified to date, G9a (EHMT2) and EZH2 have emerged as key epigenetic regulators of tumor progression owing to their central roles in establishing H3K9me2- and H3K27me3-mediated transcriptional repression. Their coordinated activities contribute to oncogenic transcriptional programs, cellular plasticity, immune evasion, and therapeutic resistance across multiple malignancies, including melanoma.

## 4 G9a (EHMT2) in cutaneous melanoma

Cutaneous melanoma is highly dependent on epigenetic adaptation mechanisms. Despite advances in targeted therapy with BRAF/MEK inhibitors and immunotherapy (anti–PD-1/PD-L1, anti-CTLA-4, and anti-LAG3), many patients develop adaptive resistance through phenotypic dedifferentiation, transcriptional reprogramming, and immune response gene silencing, sustained by repressive histone marks, particularly H3K9me2 (catalyzed by G9a/EHMT2 and GLP/EHMT1) and H3K27me3 (catalyzed by EZH2) (Flesher and Fisher, 2021).

**FIGURE 2:**
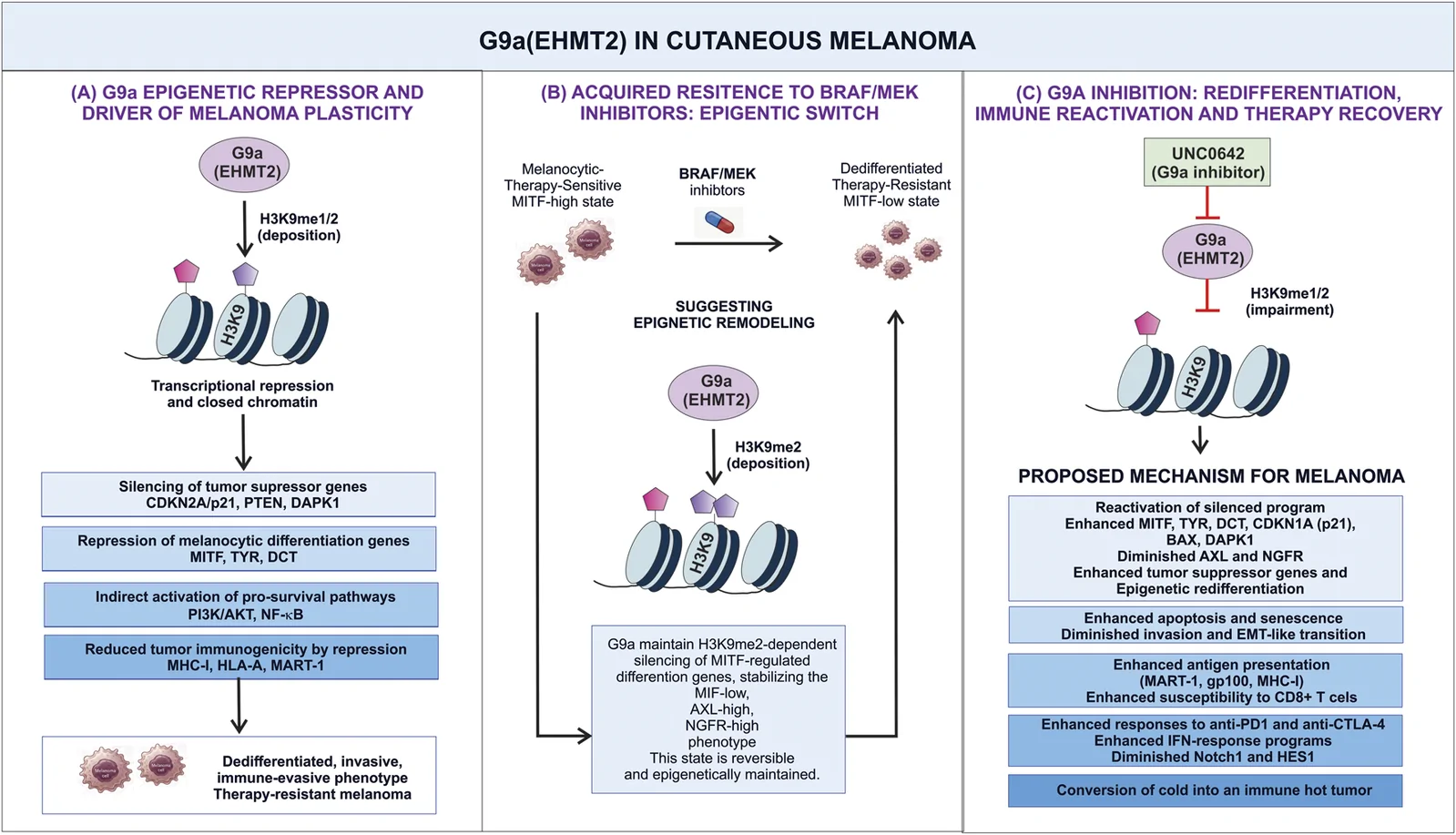
Central role of G9a (EHMT2) in melanoma plasticity, therapeutic resistance, and immune evasion. **(A)** G9a-mediated H3K9me1/2 deposition promotes chromatin compaction and transcriptional repression, resulting in silencing of tumor suppressor and melanocytic differentiation genes, activation of pro-survival pathways, reduced tumor immunogenicity, and acquisition of a dedifferentiated, therapy-resistant phenotype. **(B)** During acquired resistance to BRAF/MEK inhibitors, melanoma undergoes reversible epigenetic reprogramming from a MITF-high melanocytic state to a MITF-low/AXL-high/NGFR-high dedifferentiated state, driven by increased H3K9me2 and H3K27me3 deposition and associated with enhanced plasticity, invasion, immune evasion, and therapeutic resistance. **(C)** Proposed mechanism of G9a inhibition in melanoma. Based on preclinical evidence and the known biological functions of G9a, pharmacological inhibition with UNC0642 is proposed to reduce H3K9me2 levels, promote epigenetic redifferentiation, restore tumor suppressor and melanocytic gene expression, enhance antigen presentation and antitumor immunity, and improve responsiveness to MAPK-targeted therapy and immune checkpoint blockade. These effects represent a mechanistic model derived from available preclinical studies and related experimental evidence and have not yet been fully validated in melanoma.

G9a is a histone methyltransferase that catalyzes mono- and di-methylation of histone H3 lysine 9, repressing transcription and stabilizing closed chromatin states. It promotes silencing of tumor suppressor genes (CDKN2A/p21, PTEN, DAPK1), repression of melanocytic differentiation genes (including MITF, TYR, DCT), indirect activation of pro-survival pathways (PI3K/AKT, NF-κB), and reduction of tumor immunogenicity through the repression of melanoma antigens and antigen-presentation genes, including MHC-I, HLA-A, and MART-1 repression. This creates a dedifferentiated, invasive phenotype characteristic of therapy-resistant melanomas, positioning G9a as an epigenetic maintainer of tumor plasticity (Figure 2A) (Katayama et al., 2020; Jan et al., 2021).

A hallmark of acquired resistance to BRAF/MEK inhibitors is the reversible transition from a melanocytic, MITF-high state to a dedifferentiated phenotype characterized by low MITF expression and increased levels of AXL and nerve growth factor receptor (NGFR/CD271) (Hartman et al., 2020; Huang et al., 2021). This transcriptional switch is accompanied by widespread epigenetic remodeling, including increased deposition of H3K9me2 and H3K27me3, which stabilize the repression of melanocytic lineage genes while promoting the expression of invasive, mesenchymal-like, and neural crest-associated transcriptional programs (Flesher and Fisher, 2021; Huang et al., 2021). G9a contributes to this process by maintaining H3K9me2-dependent silencing of MITF-regulated differentiation genes, whereas the resulting MITF-low/AXL-high/NGFR-high phenotype exhibits enhanced cellular plasticity, invasiveness, stem-like properties, and intrinsic resistance to MAPK-targeted therapy (Hartman et al., 2020). Importantly, because this phenotype is epigenetically maintained rather than genetically fixed, pharmacological inhibition of G9a may promote redifferentiation and restore sensitivity to BRAF/MEK inhibitors (Figure 2B).

UNC0642 is a second-generation G9a inhibitor with high potency, oral bioavailability, and plasma stability that reduces H3K9me2 levels and reactivates silenced genes (Kelly et al., 2021). Pharmacological G9a inhibition disrupts cooperative regulatory networks, particularly with EZH2, resulting in synergistic gene derepression when combined with Tazemetostat. Dual targeting strategies such as CM-272 (G9a/DNMT1 inhibitor) have demonstrated significant antitumor activity in murine melanoma models (Nylund et al., 2025; De Beck et al., 2022).

Experimental studies demonstrate that UNC0642 suppresses melanoma cell proliferation, migration, and invasion, induces apoptosis, reduces H3K9me2 deposition, and inhibits Notch1/HES1 signaling, resulting in significant inhibition of melanoma growth both *in vitro* and *in vivo* (Dang et al., 2020). Furthermore, G9a inhibition enhances the efficacy of immune checkpoint blockade by increasing tumor immunogenicity, promoting LC3B-associated autophagy, and enhancing CD8^+^ T-cell infiltration (Kelly et al., 2021). Collectively, these findings suggest that pharmacological inhibition of G9a may facilitate epigenetic reprogramming toward a more differentiated and immunogenic phenotype, although direct restoration of the MITF-high/AXL-low state remains to be demonstrated experimentally in melanoma (Figure 2C).

### 4.1 Preclinical and translational development of G9a inhibitors

Although G9a/EHMT2 has emerged as a compelling epigenetic target in cancer, selective G9a/GLP inhibitors have not yet progressed into clinical evaluation and remain largely at the preclinical stage, owing to challenges associated with toxicity, suboptimal pharmacokinetic properties, limited bioavailability, and the need for improved target selectivity (Silva-Carvalho et al., 2024; He et al., 2025).

Early compounds such as BIX-01294 provided the first proof-of-concept inhibitors of G9a/GLP activity. BIX-01294 reduces H3K9 methylation and has demonstrated anticancer effects in several tumor models, including inhibition of proliferation, induction of apoptosis, and modulation of autophagy-related pathways. However, its clinical translation has been limited by cytotoxicity and suboptimal pharmacological properties (Vedadi et al., 2011; Ni et al., 2024).

Second-generation inhibitors, including UNC0638 and UNC0642, were developed to improve potency and selectivity. UNC0638 shows potent *in vitro* G9a/GLP inhibitory activity but has poor pharmacokinetic properties, limiting its use in animal studies. In contrast, UNC0642 displays improved pharmacokinetics, higher selectivity, and lower cytotoxicity, making it more suitable for *in vivo* preclinical evaluation (Vedadi et al., 2011; Ni et al., 2024).

In melanoma, preclinical evidence supports G9a as a promising therapeutic target. G9a is overexpressed in melanoma cells, and pharmacological inhibition with UNC0642 has been shown to suppress melanoma cell growth and induce apoptosis, at least in part through modulation of the Notch1 signaling pathway. Importantly, G9a inhibition may also enhance antitumor immunity. Preclinical melanoma studies indicate that G9a blockade can sensitize tumors to immune checkpoint blockade leading to increased melanoma cell death and induction of LC3B-associated autophagy pathways. Higher LC3B expression has been associated with improved response to checkpoint inhibition, suggesting its potential utility as a predictive biomarker (Dang et al., 2020; Kelly et al., 2021).

The role of G9a in tumor immune exclusion has been further clarified in a recent study by Adan-Villaescusa and colleagues in hepatocellular carcinoma, even though not yet validated in melanoma settings. These findings are discussed because they reveal conserved epigenetic mechanisms of immune evasion that may be relevant to melanoma biology, their applicability to melanoma remains to be experimentally confirmed. Using two distinct G9A inhibitors, CM272 and EZM8266, the authors demonstrated that G9a inhibition reduced H3K9me2 enrichment at the promoters of CXCL10 and class I MHC genes, restored surface MHC-I expression, and enhanced IFN-γ driven CXCL10 secretion. They also showed that G9a promoted the de-repression of endogenous retroviral elements, accumulation of intracellular double-stranded RNA, and activation of interferon-responsive innate immune programs, thereby further enhancing tumor immunogenicity, representing a mechanism of innate immune sensing not previously described for G9a in solid tumors (Adan-Villaescusa et al., 2026).

In murine models, both inhibitors synergized with anti-PD-1 to produce near-complete tumor regression, accompanied by significantly increased CD8^+^ T cell infiltration, reduced Tregs and reprograming of tumor-associated macrophages toward an inflammatory, antigen-presenting phenotype (Adan-Villaescusa et al., 2026).

Although these findings were obtained in hepatocellular carcinoma, they present a conserved mechanism of G9a-mediated immune evasion through suppression of antigen presentation, T cell recruitment and innate immune signaling, processes highly relevant to the immune-excluded phenotype observed in melanoma.

Together, these findings indicate that G9a inhibitors remain at the preclinical stage, but collectively provide a strong rationale for future clinical development, particularly in combination with immunotherapy. In melanoma, the most promising translational direction may involve using G9a inhibition to reprogram tumor cell-intrinsic epigenetic states, enhance tumor immunogenicity, and overcome resistance to immune checkpoint blockade.

## 5 Structure and function of EZH2

**FIGURE 3:**
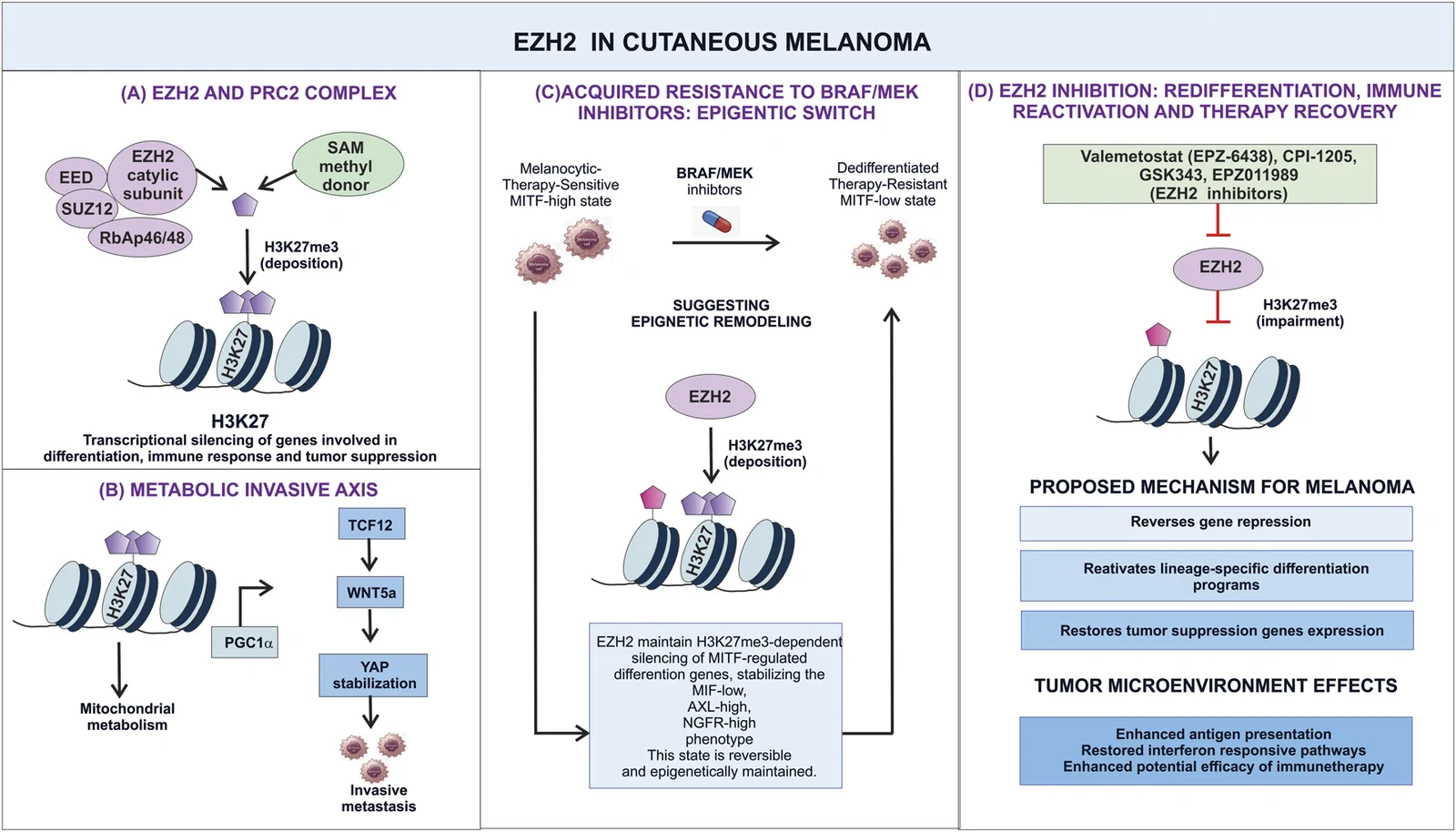
EZH2-mediated epigenetic regulation in cutaneous melanoma. **(A)** EZH2, the catalytic subunit of the Polycomb Repressive Complex 2 (PRC2), catalyzes H3K27 trimethylation (H3K27me3), leading to transcriptional repression of genes involved in melanocytic differentiation, tumor suppression, and immune regulation. **(B)** EZH2 promotes melanoma invasion and metastasis through repression of PGC1α and activation of the TCF12–WNT5A–YAP signaling axis. **(C)** During acquired resistance to BRAF/MEK inhibitors, increased EZH2 activity promotes H3K27me3-dependent epigenetic remodeling, driving reversible phenotype switching from a MITF-high melanocytic state to a dedifferentiated MITF-low/AXL-high/NGFR-high state associated with therapeutic resistance and immune evasion. **(D)** Pharmacological inhibition of EZH2 reduces H3K27me3 levels, restores lineage-specific differentiation and tumor suppressor gene expression, and enhances antigen presentation and interferon-responsive pathways, thereby promoting tumor redifferentiation and improving the potential efficacy of targeted therapy and immunotherapy. The mechanisms depicted in panels **(C)** and **(D)** represent proposed models based on current preclinical evidence and have not yet been fully validated in cutaneous melanoma.

EZH2 is the catalytic subunit of the Polycomb Repressive Complex 2 (PRC2), which includes EED, SUZ12, and RbAp46/48. Using S-adenosylmethionine (SAM) as a methyl donor, EZH2 promotes trimethylation of histone H3 at lysine 27 (H3K27me3), leading to transcriptional silencing of genes involved in differentiation, immune response, and tumor suppression (Figure 3A) (Fisher et al., 2016; Chen et al., 2018). Mechanistically, EZH2 does not act in isolation but cooperates with other epigenetic modifiers, including DNMT1 and G9a, to establish and maintain a repressive chromatin landscape that reinforces transcriptional programs associated with tumor stemness, cellular plasticity, and therapeutic resistance (Casciello et al., 2022).

During normal neural crest development, EZH2 temporarily represses differentiation genes (e.g., MITF, DCT, and TYR) to maintain the balance between proliferation and differentiation in melanocytic precursors. Upon differentiation, EZH2 activity naturally decreases, thereby establishing stable, non-proliferative melanocytes. However, in melanoma, this regulatory control is lost, transforming EZH2 into a pathological driver of tumor plasticity that enables cells to transition between proliferative and invasive states, an adaptive feature associated with metastasis and immune evasion (Dilshat et al., 2021; Tigu et al., 2025).

EZH2 overexpression occurs in approximately 60%–70% of metastatic melanomas, correlating with increased tumor thickness, lymph node metastasis, therapy resistance, and poor survival. Activation occurs through gain-of-function mutations, most notably the Y641 and A677G hotspot mutations, which enhance H3K27 trimethylation activity, or indirectly via BRAF/MEK/ERK-mediated phosphorylation at S21, which modulates target gene selectivity and enables phenotypic switching (Zhou et al., 2025; Verma et al., 2025).

In BRAF inhibitor-resistant melanomas, elevated EZH2 expression is maintained. Combined BRAF and EZH2 inhibition restores vemurafenib sensitivity, reduces cell viability, induces cell cycle arrest, and increases apoptosis. This combination suppresses mitosis-related pathways and significantly reduces the expression of PLK1, a key downstream target whose high expression correlates with worse survival in BRAF-mutant patients (Uebel et al., 2023).

Beyond transcriptional repression, EZH2 regulates melanoma metastasis through a metabolic-invasive axis. EZH2 silences PGC1α via H3K27me3 deposition, reducing mitochondrial metabolism while promoting invasion through TCF12-mediated WNT5A activation and subsequent YAP stabilization. EZH2 inhibition restores PGC1α and suppresses this metastatic program (Figure 3C) (Luo et al., 2020).

Importantly, EZH2 also contributes to the epigenetic regulation of melanoma phenotypic plasticity by controlling the balance between the melanocytic MITF-high state and the dedifferentiated, therapy-resistant AXL-high/NGFR-high state (Tigu et al., 2025).

Importantly, EZH2 contributes to melanoma phenotypic plasticity by epigenetically regulating the balance between the differentiated MITF-high melanocytic state and the dedifferentiated, therapy-resistant AXL-high/NGFR-high state. During prolonged BRAF/MEK inhibition, increased EZH2 activity remodels the chromatin landscape through H3K27me3 deposition, repressing melanocytic lineage programs while promoting neural crest-like and mesenchymal transcriptional states characterized by elevated AXL and NGFR expression (Figure 3C). This reversible epigenetic reprogramming drives phenotype switching, enabling melanoma cells to escape MAPK inhibition, acquire invasive behavior, and develop resistance to targeted therapies (Perotti et al., 2019).

Moreover, the MITF-low/AXL-high phenotype is associated with reduced antigen presentation, impaired T-cell recognition, and an immunologically “cold” tumor microenvironment, further linking EZH2-mediated chromatin remodeling to both therapeutic resistance and immune evasion (Benboubker al., 2022). Preclinical studies further demonstrate that pharmacological inhibition of EZH2 suppresses invasive transcriptional programs, restores melanocytic differentiation markers including MITF, reduces AXL-associated dedifferentiation signatures, and enhances sensitivity to MAPK-targeted therapies, supporting EZH2 as an attractive strategy to overcome acquired resistance (Perotti et al., 2019).

NRAS-mutant melanomas exhibit unique epigenetic reprogramming characterized by bivalent H3K27me3/H3K4me3 marks and broad H3K4me3 domains regulating invasive genes (including ZEB1, TWIST1, and SOX9). EZH2/PRC2 inhibition disrupts these chromatin states, reducing invasiveness with minimal effects on cell proliferation. Preclinical combination therapy with MEK inhibitors significantly reduces tumor burden, highlighting PRC2-mediated epigenetic reprogramming as a specific therapeutic vulnerability (Terranova, 2021).

Emerging epigenetic therapies targeting EZH2 have gained increasing attention, with next-generation inhibitors such as valemetostat showing encouraging clinical and preclinical activity (Tachibana et al., 2024). Selective EZH2 inhibitors, including CPI-1205, GSK343, and EPZ011989, have demonstrated promising preclinical efficacy by reversing H3K27me3-mediated gene repression, reactivating lineage-specific differentiation programs, and promoting a more differentiated cellular phenotype (Figure 3D) (Tigu et al., 2025; Resch et al., 2025). These agents also modulate the tumor microenvironment, enhancing antigen presentation, restoring interferon-responsive pathways, and thereby increasing the potential efficacy of immunotherapy.

### 5.1 EZH2 as a regulator of tumor immunity and immune checkpoint synergy

EZH2 hyperactivity promotes immune evasion by suppressing antigen presentation, interferon signaling, genes involved in T-cell recruitment through H3K27me3 deposition (Goswami et al., 2018). During immunotherapy, increased T-cell infiltration and TNF-α production paradoxically induce EZH2 overexpression, which in turn silences MHC class I, CXCL9/CXCL10, and melanocytic antigens, thereby promoting tumor dedifferentiation and immunosuppression. Conversely, EZH2 inhibition restores antigen presentation, decreases PD-L1, and enhances CD8^+^ T-cell infiltration, demonstrating marked therapeutic synergy with immune checkpoint blockade in preclinical murine models (Zingg et al., 2017).

Tazemetostat, an FDA-approved EZH2 inhibitor for epithelioid sarcoma and follicular lymphoma, has demonstrated limited melanoma efficacy as a monotherapy in melanoma, but shows considerable promise in combination-based therapeutic strategies (Hoy, 2020; Oppelt et al., 2025; Yi et al., 2024). EZH2 cooperates with chromatin-remodeling factors such as Mi-2β to suppress interferon-stimulated genes, whereas dual inhibition of these pathways restores chemokine expression and improves anti-PD-1 responses (Li et al., 2024).

In addition, PRC2 suppresses IFN-induced MHC class II expression via H3K27me3 at the CIITA promoter, thereby reducing antigen presentation and contributing to resistance to PD-1 blockade. Pharmacological EZH2 inhibition restores chromatin accessibility at the CIITA locus leading to re-expression of MHC-II (James et al., 2023). Furthermore, CRISPR-based functional screens have demonstrated that EZH2 inhibition restores MHC expression, chemokines, T and NK cell mediated cytotoxicity, highlighting its potential to enhance antitumor immunity (Kato et al., 2023).

Beyond tumor cells, EZH2 modulates the immune microenvironment. For example, CRISPR/dCas9-mediated HIF1A silencing in tumor-associated macrophages enhances phagocytosis and CD8^+^ T-cell responses (Dong et al., 2021; Hou et al., 2025). Moreover, EZH2 also epigenetically represses the STING pathway; pharmacological inhibition of EZH2 restores STING expression and reactivates type I interferon signaling, further potentiating antitumor immune responses (Xu et al., 2022).

Potential biomarkers predictive of response to EZH2 inhibition, include baseline EZH2/H3K27me3 levels, interferon signatures, expression of antigen-presentation machinery (including MHC pathways), and chromatin alterations such as INI1/SMARCB1 loss (Chi et al., 2023; He and Wu, 2025). To further improve therapeutic efficacy and overcome acquired resistance, combination strategies involving DOT1L inhibitors, immune checkpoint blockade, or BRAF/MEK therapies are currently being investigated (Kazansky et al., 2024; DuCote et al., 2024).

### 5.2 Clinical trials of EZH2 inhibitors in solid tumors

Although clinical development of EZH2 inhibitors has advanced considerably across several solid tumors and hematologic malignancies, melanoma-specific clinical evidence remains limited. Nevertheless, these studies provide important translational insights because many of the biological mechanisms targeted by EZH2 inhibition including repression of MHC-I antigen presentation, interferon signaling, chemokine expression, and regulation of tumor phenotypic plasticity, are highly relevant to melanoma progression, immune evasion, and therapeutic resistance, which will be further discussed in the next section. Therefore, the clinical experience obtained from other solid tumors provides a valuable framework for the rational development of future biomarker-driven and combination strategies in melanoma.

#### 5.2.1 EPZ class inhibitors and tazemetostat

Within the EPZ class, tazemetostat has emerged as the most clinically advanced EZH2 inhibitor. Early Phase I/II basket trials evaluated tazemetostat across a range of advanced solid tumors, including those characterized by deficiencies in chromatin remodeling complexes, such as INI1 (SMARCB1) deficient and SMARCA4 deficient malignancies. More recent studies, including NCT05023655, have focused on ARID1A-mutated solid tumors, leveraging a synthetic lethality framework to enhance therapeutic efficacy. These trials have demonstrated that the clinical activity of EZH2 inhibition is highly dependent on tumor genotype, with the most robust responses observed in tumors harboring specific epigenetic vulnerabilities. Collectively, these findings establish Tazemetostat as a paradigm for biomarker-driven epigenetic therapy in solid tumors (Italiano et al., 2018).

#### 5.2.2 Valemetostat (dual EZH1/2 inhibitor)

Clinical development of valemetostat represents a shift toward broader epigenetic targeting through simultaneous inhibition of EZH2 and its homolog, EZH1. In solid tumors, early-phase studies are ongoing, including the Phase Ib trial NCT05633979, which evaluates valemetostat in combination with trastuzumab deruxtecan in patients with HER2-low or HER2-ultralow metastatic breast cancer. This study reflects a growing emphasis on combinatorial strategies, as dual EZH1/2 inhibition is hypothesized to enhance chromatin reprogramming and overcome compensatory mechanisms that may limit the efficacy of EZH2-selective agents. Although these studies remain in the early stages, they suggest that valemetostat may have broader applicability across solid tumors, particularly when used to potentiate targeted therapies (Iwase et al., 2024; Choudhury et al., 2024).

#### 5.2.3 CPI-1205 (lirametostat)

The clinical evaluation of CPI-1205 began with first-in-human Phase I trials enrolling patients with advanced solid tumors and lymphomas. These studies established its safety profile, pharmacokinetics, and pharmacodynamic evidence of target engagement, including decreased H3K27me3 levels in tumor tissues. While antitumor activity as a single agent was limited in unselected solid tumors, CPI-1205 has been further explored in biomarker-driven and combination settings, particularly in prostate cancer. These early trials were instrumental in validating EZH2 as a druggable epigenetic target, while simultaneously highlighting the need for more refined patient selection and combination strategies (Yap et al., 2019).

#### 5.2.4 GSK-class EZH2 inhibitors (GSK2816126 and related compounds)

Among the earliest compounds to enter clinical testing were GSK-derived inhibitors, including GSK2816126, which was evaluated in a Phase I trial involving patients with relapsed or refractory lymphomas and advanced solid tumors. This study provided important insights into dosing, tolerability, and biological activity, although clinical responses were modest. Related compounds such as GSK126 remained largely within the preclinical or translational domain but played a critical role in demonstrating the therapeutic potential of EZH2 inhibition. Collectively, these agents established the foundational proof-of-concept that pharmacologic targeting of the PRC2 complex is feasible in human cancers, paving the way for the development of more potent and selective inhibitors (Yap et al., 2019).

#### 5.2.5 Next-generation EZH2 inhibitors and emerging trials

The field continues to evolve with the development of next-generation EZH2 inhibitors, such as tulmimetostat and other investigational compounds, which are currently being evaluated in early-phase trials across multiple solid tumor types. These studies increasingly incorporate biomarker stratification, including ARID1A mutations, SMARCB1 loss, and other indicators of PRC2 dependency, together with rational combination strategies involving immunotherapy and targeted agents. Overall, these next-generation clinical studies reflect an increasingly sophisticated understanding of EZH2 biology, positioning epigenetic therapy not as an isolated cytotoxic strategy but as a means of reprogramming tumor phenotype, restoring antitumor immunity, and enhancing responsiveness to complementary therapeutic modalities.

### 5.3 Relevance to melanoma

Despite the expanding clinical investigation of EZH2 inhibitors in multiple solid tumors, melanoma remains relatively underrepresented in these trials. This underrepresentation likely reflects the absence of recurrent activating EZH2 mutations in melanoma, in contrast to other malignancies perticularly B cell lymphomas, where these alterations are more prevalent (Kim and Roberts, 2016; Italiano et al., 2018). Nevertheless, the limited clinical evidence should not be interpreted as a lack of biological relevance. Instead, the rationale for targeting EZH2 in melanoma is supported by its well-established role in epigenetic reprogramming, tumor plasticity, and immune escape, rather than by recurrent genomic alterations.

EZH2 serves as a central regulator of melanoma progression by maintaining dedifferentiated, invasive tumor states and promoting immune evasion through H3K27me3-mediated repression of antigen presentation, interferon signaling, and immune-related gene expression (Zingg et al., 2015; Zingg et al., 2017; Tiffen et al., 2015). Its activity contributes to the transition from a melanocytic, therapy-sensitive phenotype to a dedifferentiated, invasive, and therapy-resistant state, highlighting its pivotal role in melanoma phenotypic plasticity (Tiffen et al., 2016).

Consistent with this mechanism, preclinical studies have demonstrated that EZH2 inhibition can restore tumor immunogenicity, reactivates major histocompatibility complex (MHC) class I and II expression, increases chemokine production, and enhances CD8^+^ T-cell infiltration into the tumor microenvironmen (Zingg et al., 2017).

Furthermore, EZH2 blockade reactivates interferon signaling, improves antigen processing and presentation, and promotes a more inflamed tumor microenvironment, thereby sensitizing tumors to immune-mediated clearance (Zhou et al., 2021; Guo et al., 2020).

Collectively, these findings provide the biological basis for translating the encouraging results observed in other solid tumors to melanoma, despite the current lack of melanoma-specific clinical trials. Consequently, the therapeutic potential of EZH2 inhibitors in melanoma is expected to lie primarily in rational combination strategies rather than as standalone agents. Combining EZH2 inhibition with immune checkpoint blockade, BRAF/MEK-targeted therapies, or adoptive cell therapies such as tumor-infiltrating lymphocytes (TILs) may simultaneously reverse epigenetic silencing, restore tumor immunogenicity, and overcome therapy-induced phenotypic plasticity (Zingg et al., 2017).

Overall, EZH2 inhibition should be viewed not only as a direct anti-tumor strategy but also as an epigenetic immunomodulatory approach capable of reprogramming the melanoma microenvironment to enhance responsiveness to existing therapies. This concept provides a strong translational framework for future melanoma-specific clinical studies evaluating EZH2-based combination therapies, particularly in patients with dedifferentiated, immune-cold, or therapy-resistant disease (Tiffen et al., 2015).

## 6 Crosstalk between G9a and EZH2

### 6.1 Redundant and cooperative repression mechanisms

**FIGURE 4:**
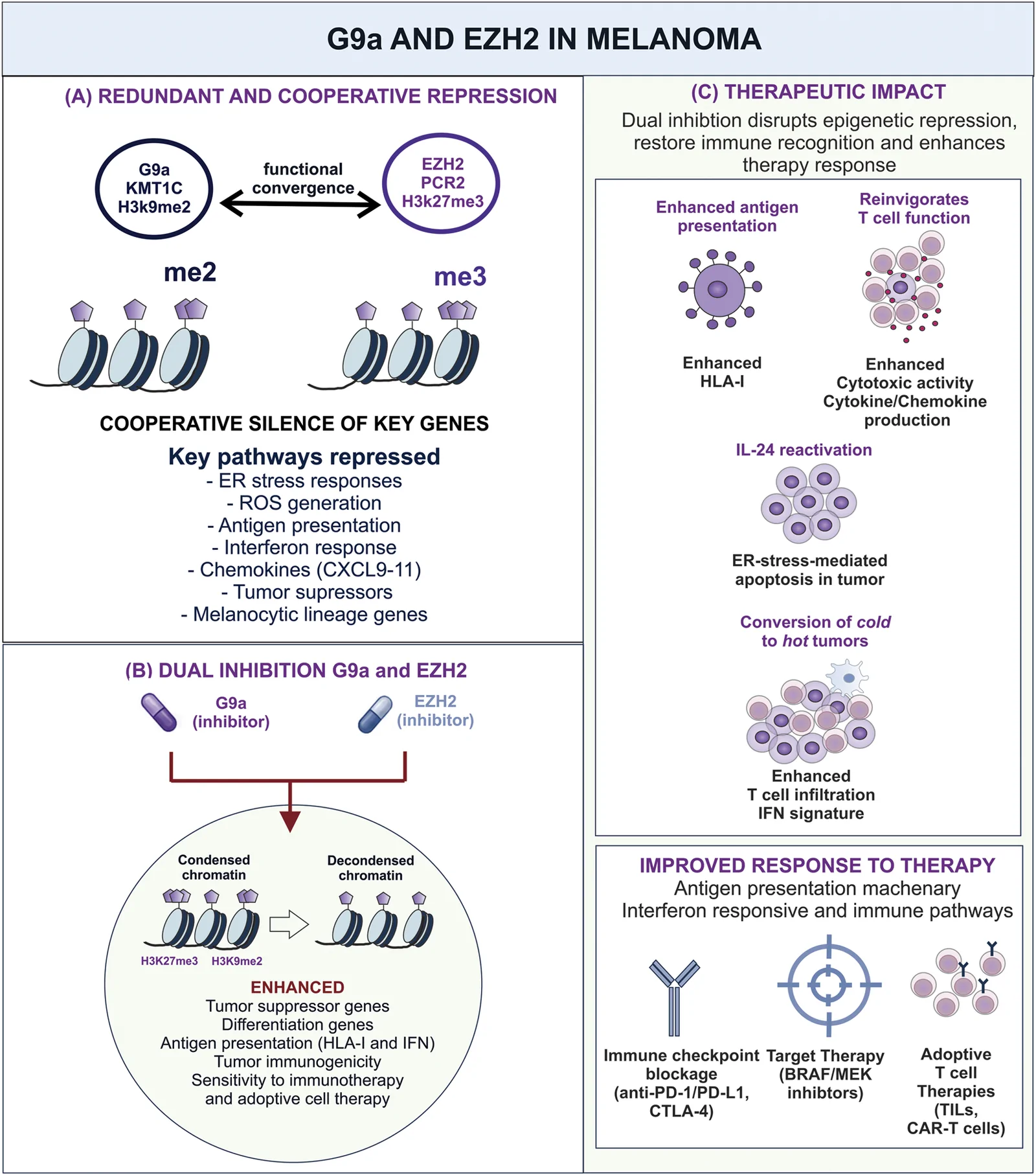
Cooperative epigenetic regulation by G9a and EZH2 in melanoma. **(A)** G9a (EHMT2)-mediated H3K9me2 and EZH2/PRC2-mediated H3K27me3 cooperate to establish stable transcriptional repression of genes involved in antigen presentation, interferon signaling, chemokine production, ER stress responses, tumor suppression, and melanocytic differentiation, thereby promoting melanoma plasticity, immune evasion, and therapeutic resistance. **(B)** Simultaneous inhibition of G9a and EZH2 disrupts this repressive chromatin state, restoring expression of tumor suppressor, differentiation, and immune-related genes while increasing tumor immunogenicity. **(C)** Dual epigenetic targeting enhances antigen presentation, T-cell activation, IL-24-mediated ER stress-induced apoptosis, and conversion of immune-“cold” tumors into immune-“hot” tumors, improving the efficacy of immune checkpoint blockade, targeted therapies, and adoptive T-cell therapies. Abbreviations: ER, endoplasmic reticulum; H3K9me2, histone H3 lysine 9 dimethylation; H3K27me3, histone H3 lysine 27 trimethylation; IFN, interferon; IL-24, interleukin-24; MHC-I, major histocompatibility complex class I; PRC2, Polycomb Repressive Complex 2; ROS, reactive oxygen species; TILs, tumor-infiltrating lymphocytes.

Although G9a and EZH2 catalyze distinct histone modifications, namely H3K9me2 and H3K27me3, respectively, they converge functionally to establish and maintain transcriptional silencing. This functional convergence extends beyond chromatin remodeling and encompasses the coordinated regulation of multiple oncogenic pathways. G9a and EZH2 are frequently overexpressed across diverse cancer types and cooperate to repress genes involved in endoplasmic reticulum (ER) stress responses and reactive oxygen species (ROS) generation. Notably, simultaneous inhibition of both enzymes induces pronounced tumor cell death through reactivation of interleukin-24 (IL-24), a tumor suppressor cytokine that selectively triggers apoptosis in malignant cells while sparing normal tissues. These findings indicate that G9a and EZH2 cooperate to enable tumor cells to evade ER stress-mediated apoptosis by epigenetically suppressing IL-24 expression (Figure 4A) (Casciello et al., 2022).

In addition to its functional interaction with EZH2, G9a cooperates with DNA methylation machinery, particularly DNA methyltransferase 1 (DNMT1), thereby linking histone and DNA methylation to reinforce stable transcriptional repression. Furthermore, G9a intersects with oncogenic signaling pathways, including Notch signaling, contributing to the maintenance of cancer stem-like properties and reinforcing repression of melanocytic lineage-specific regulators, processes that are particularly relevant to melanoma dedifferentiation and therapeutic resistance (Shi et al., 2024; Dang et al., 2020).

The functional redundancy between G9a and EZH2 relies on their ability to silence many genomic loci, particularly tumor suppressor genes and immune-related genes, through complementary repressive histone modifications. As a result, inhibiting one enzyme often leads to only partial gene reactivation, because the remaining methyltransferase can compensate for the absent one and maintain transcriptional repression.

Cooperative behavior is further supported by evidence demonstrating that H3K9me2-marked regions can facilitate recruitment of PRC2, while G9a activity helps establish chromatin microenvironments permissive to PRC2 occupancy. And both systems contribute to maintaining long-term silenced chromatin states. Together, G9a and EZH2 establish a robust and resilient epigenetic network that maintains long-term transcriptional silencing and is therefore difficult to reverse through single-agent inhibition (Mozzetta et al., 2014).

### 6.2 H3K9me2–H3K27me3 interplay

The relationship between these marks is dynamic and hierarchical rather than simply additive. H3K9me2 often associated with early or intermediate stages of facultative heterochromatin and can act as a nucleation signal for repressive chromatin domains. In contrast, H3K27me3 is associated with stable, yet potentially reversible, gene repression and, plays a key role in lineage-specific transcriptional control (Margueron and Reinberg, 2011).

The functional interplay between these modifications is based on the ability of H3K9me2-enriched regions to facilitate PRC2 recruitment and spreading. Once recruited, PRC2 can extend transcriptional repression across broader genomic regions, whereas the co-occupancy of H3K9me2 and H3K27me3 results in deeply silenced, transcriptionally inert chromatin domains (Margueron and Reinberg, 2011).

In melanoma, this dual epigenetic marking is frequently observed at gene involved in antigen presentation pathways (e.g., MHC class I genes), interferon response programs and chemokines involved in T cell recruitment thereby contributing to immune evasion and resistance to immunotherapy (Figure 4A).

### 6.3 Chromatin compaction synergy

One of the most consequential outcomes of the crosstalk between G9a and EZH2 is the establishment of a synergistic program of chromatin compaction. G9a-mediated deposition of H3K9me2 promotes the recruitment of heterochromatin protein 1 (HP1), which is central to the formation of condensed chromatin structures. In parallel, EZH2 catalyzes H3K27me3, leading to the recruitment of PRC1 complexes and subsequent ubiquitination of H2AK119, a modification that further reinforces chromatin compaction. When these two pathways operate simultaneously, their effects are not merely additive but highly synergistic, driving chromatin into a densely compacted and transcriptionally inaccessible state. Under these conditions, the binding of transcription factors is severely impaired, and affected loci become stably and heritably repressed (Kundu et al., 2017).

### 6.4 Combined inhibition strategies

Functionally, this level of chromatin organization has direct implications for tumor biology. It contributes to the emergence of immune-excluded or “immune-cold” tumor phenotypes, characterized by diminished expression of antigen presentation machinery, reduced production of inflammatory mediators, and limited recruitment of effector immune cells. As a result, tumor immunogenicity is significantly compromised, and resistance to immunotherapeutic approaches, including immune checkpoint blockade, is reinforced (Figure 4B).

Moreover, the cooperative regulation of ER stress pathways by G9a and EZH2 provides an additional rationale for dual epigenetic targeting. Simultaneous inhibition of both enzymes reactivates IL-24 expression, promotes ER stress-mediated apoptosis, and selectively induces tumor cell death, further supporting the therapeutic advantages of combined inhibition over single-agent approaches (Casciello et al., 2022).

The mechanistic framework provides a strong rationale for combined inhibition strategies targeting both G9a and EZH2. Inhibition of EZH2 alone often fails to fully restore gene expression because H3K9me2-dependent repression persists, while inhibition of G9a alone leaves PRC2-mediated silencing intact. This creates a form of epigenetic compensation, allowing tumor cells to maintain transcriptional repression despite single-agent intervention.

In contrast, dual inhibition of G9a and EZH2 has the potential to more comprehensively dismantle these repressive epigenetic networks, leading to robust reactivation of previously silenced genes. This includes the restoration of antigen presentation pathways such as MHC class I, the re-expression of interferon-stimulated genes, and the induction of chemokines including CXCL9, CXCL10, and CXCL11 which are critical for T cell recruitment (Figure 4C).

Consequently, the tumor cells become more immunogenic and permissive to immune infiltration, enhancing both the presence and functional capacity of tumor-infiltrating lymphocytes. Current therapeutic strategies under investigation include combinaing G9a inhibitors, such as UNC0638 or BIX-01294, with EZH2 inhibitors like tazemetostat and CPI-1205. These approaches are also being integrated with immunotherapeutic modalities, including anti-PD-1 checkpoint blockade and adoptive T-cell therapies based on tumor-infiltrating lymphocytes (TILs), with the aim of exploiting the increased immune visibility of tumor cells following epigenetic reprogramming (Kelly et al., 2021; Liang et al., 2023; Liguori et al., 2025).

Conceptually, the G9a–EZH2 axis can be understood as a dual-layer epigenetic lock. G9a establishes the initial repressive chromatin landscape, while EZH2 reinforces and propagates this state across the genome, ensuring its stability and heritability. Disrupting only one component of this system is often insufficient to reverse gene silencing Conversely, simultaneous targeting of both layers has the potential to reprogram tumor cells toward a more transcriptionally active and immunologically responsive state, thereby providing a compelling therapeutic strategy to overcome tumor plasticity, immune evasion, and treatment resistance in melanoma.

## 7 Challenges and future perspectives

Despite a strong biological rationale, the clinical translation of G9a and EZH2 inhibition in melanoma remains limited by several critical challenges. Issues of selectivity and toxicity are paramount, as currently available compounds frequently exhibit off-target effects and suboptimal pharmacokinetic profiles, thereby constraining dose intensity and long-term therapeutic efficacy. Given the fundamental roles of epigenetic regulators in normal cellular homeostasis, systemic inhibition also poses a significant risk of immune and hematopoietic toxicity. In addition, functional redundancy within epigenetic networks, particularly between G9a- and PRC2-mediated repression, attenuates the efficacy of single-agent approaches and underscores the need for rational combination strategies. In this context, the development of next-generation inhibitors guided by predictive biomarkers will be essential to enhance therapeutic precision and clinical benefit.

An additional challenge is the dual, or “double-edged sword,” effect of epigenetic therapies on the tumor immune microenvironment. Although inhibition of G9a and EZH2 enhances tumor immunogenicity by restoring antigen presentation, interferon signaling, and chemokine expression, these enzymes also play fundamental roles in the differentiation, maintenance, and function of immune cells. EZH2 is required for the maintenance of stem-like and memory CD8^+^ T-cell populations, which are critical for durable anti-tumor immunity and sustained responses to immune checkpoint blockade and adoptive cell therapies (Goswami et al., 2018; Hou et al., 2025). Consequently, prolonged systemic EZH2 inhibition may inadvertently impair T-cell persistence, proliferative capacity, and cytotoxic function, potentially limiting long-term therapeutic efficacy despite increasing tumor immunogenicity.

Beyond effector T cells, systemic epigenetic inhibition may also influence immunosuppressive populations within the tumor microenvironment. EZH2 contributes to the stability and suppressive function of regulatory T cells, while epigenetic remodeling can alter the recruitment, differentiation, and activity of myeloid-derived suppressor cells and tumor-associated macrophages. The overall therapeutic outcome is therefore likely to depend on the balance between enhanced tumor susceptibility to immune attack and the simultaneous effects on both effector and suppressive immune populations. A more comprehensive understanding of these context-dependent responses will be essential for optimizing the clinical application of epigenetic therapies.

A further challenge lies in the effective integration of epigenetic therapies into existing treatment paradigms, particularly in combination with immune checkpoint blockade and targeted therapies. The timing and sequencing of epigenetic modulation are likely to be critical determinants of therapeutic outcome. Transient chromatin reprogramming may enhance tumor immunogenicity and sensitize tumors to immunotherapy, whereas sustained inhibition could induce adaptive resistance mechanisms or impair immune-cell fitness and long-term immune surveillance.

The incorporation of epigenetic agents into adoptive cell therapies, including tumor-infiltrating lymphocytes (TILs) and CAR-T cells, represents a promising yet still underexplored avenue. Emerging evidence suggests that epigenetic modulation can enhance antigen presentation and improve T-cell functionality, positioning these agents as potential enablers of cellular immunotherapy.

However, this strategy will require careful optimization to maximize tumor reprogramming while minimizing detrimental effects on therapeutic lymphocytes. Potential approaches include tumor-targeted drug delivery systems, intermittent or pulse-dosing schedules that limit prolonged immune-cell exposure, and transient *ex vivo* epigenetic modulation during TIL or CAR-T cell manufacturing prior to reinfusion. Recent studies further suggest that temporally controlled EZH2 inhibition during *ex vivo* expansion may preserve T-cell stemness while enhancing antitumor function, supporting the feasibility of these approaches (Hou et al., 2025). Nevertheless, prospective validation and optimized treatment schedules remain to be established.

Future advances in the field will depend on the integration of multi-omics and single-cell technologies to resolve tumor heterogeneity and define context-specific epigenetic dependencies. These approaches hold promise for identifying predictive biomarkers, characterizing resistant subclonal populations, and refining patient stratification. Equally important will be the development of biomarkers capable of monitoring immune-cell fitness and the dynamic effects of epigenetic therapies on both tumor and immune compartments, thereby enabling treatment personalization.

In melanoma, particularly in epigenetically driven subtypes such as acral melanoma, this framework supports a shift toward precision epigenetic therapy tailored to dynamic chromatin states and the tumor immune microenvironment. Ultimately, clinical success will require mechanism-based, biomarker-informed strategies capable of modulating tumor plasticity while preserving and sustaining durable antitumor immunity.

In addition, to the cutaneous melanoma, an especially promising application of epigenetic therapies lies in acral melanoma, a biologically distinct melanoma subtype characterized by a low ultraviolet (UV)-associated mutational burden, a predominance of copy-number alterations, and an immunologically “cold” tumor microenvironment.

Unlike conventional cutaneous melanoma, which frequently relies on a high neoantigen load to elicit antitumor immune responses, acral melanoma appears to depend more heavily on epigenetic reprogramming to regulate tumor plasticity, immune evasion, and therapeutic resistance. Consequently, transcriptional repression mediated by G9a- and EZH2-dependent chromatin remodeling may play a particularly important role in silencing antigen-presentation pathways, interferon-responsive genes, and T-cell-recruiting chemokines, thereby contributing to the profound immune exclusion characteristic of this subtype. These biological features provide a strong rationale for evaluating epigenetic therapies as a means of converting acral melanoma from an immune-“cold” to a more immune-responsive phenotype. Moreover, combining G9a and/or EZH2 inhibitors with immune checkpoint blockade, adoptive cell therapies, or other immunomodulatory strategies may represent a particularly promising therapeutic approach for this historical treatment-refractory melanoma subtype. Given the limited therapeutic options and poor clinical outcomes associated with advanced acral melanoma, future biomarker-driven clinical trials specifically designed for this population should be considered a high research priority (Carvalho et al., 2023; Minowa et al., 2024; Wang et al., 2025).

## 8 Conclusion

Histone methyltransferases G9a and EZH2 are defined as central regulators of melanoma plasticity, therapeutic resistance, and immune evasion. By establishing repressive chromatin states through H3K9me2 and H3K27me3, they silence tumor suppressor genes and immune pathways, limiting antigen presentation and sustaining poorly immunogenic tumor phenotypes.

Accumulating preclinical and translational evidence supports dual targeting of G9a and EZH2 as a promising strategy to overcome redundant epigenetic repression. Combined inhibition can restore differentiation programs, enhance tumor immunogenicity, and improve responsiveness to immune checkpoint blockade and adoptive cell therapies, positioning epigenetic modulation as a means of reprogramming tumor states rather than inducing direct cytotoxicity.

Moving forward, clinical success will rely on mechanism-based combination strategies integrating epigenetic agents with immunotherapy and cellular approaches. The incorporation of predictive biomarkers, optimized treatment sequencing, and multi-omics profiling will be essential to guide patient selection and achieve durable antitumor immunity.
